# A decision-analytic method to evaluate the cost-effectiveness of remote monitoring technology for chronic depression

**DOI:** 10.1017/S0266462324004677

**Published:** 2025-01-16

**Authors:** Xiaonan Sun, Lawrence Wissow, Shan Liu

**Affiliations:** 1 Department of Industrial and Systems Engineering, University of Washington, Seattle, WA, USA; 2 Department of Psychiatry and Behavioral Sciences, University of Washington, Seattle, WA, USA

**Keywords:** cost-effectiveness analysis, depression, computer technology, treatment assessment and planning, outpatient treatment

## Abstract

**Objectives:**

Advances in mobile apps, remote sensing, and big data have enabled remote monitoring of mental health conditions, but the cost-effectiveness is unknown. This study proposed a systematic framework integrating computational tools and decision-analytic modeling to assess cost-effectiveness and guide emerging monitoring technologies development.

**Methods:**

Using a novel decision-analytic Markov-cohort model, we simulated chronic depression patients’ disease progression over 2 years, allowing treatment modifications at follow-up visits. The cost-effectiveness, from a payer’s viewpoint, of five monitoring strategies was evaluated for patients in low-, medium-, and high-risk groups: (i) remote monitoring technology scheduling follow-up visits upon detecting treatment change necessity; (ii) rule-based follow-up strategy assigning the next follow-up based on the patient’s current health state; and (iii–v) fixed frequency follow-up at two-month, four-month, and six-month intervals. Health outcomes (effects) were measured in quality-adjusted life-years (QALYs).

**Results:**

Base case results showed that remote monitoring technology is cost-effective in the three risk groups under a willingness-to-pay (WTP) threshold of U.S. GDP per capita in year 2023. Full scenario analyses showed that, compared to rule-based follow-up, remote technology is 74 percent, 67 percent, and 74 percent cost-effective in the high-risk, medium-risk, and low-risk groups, respectively, and it is cost-effective especially if the treatment is effective and if remote monitoring is highly sensitive and specific.

**Conclusions:**

Remote monitoring for chronic depression proves cost-effective and potentially cost-saving in the majority of simulated scenarios. This framework can assess emerging remote monitoring technologies and identify requirements for the technologies to be cost-effective in psychiatric and chronic care delivery.

## Introduction

Recent advances in sensors, smartphones, and wireless networks have enabled a new generation of remote healthcare monitoring technologies that promise to improve patient outcomes (1). Remote monitoring technology can potentially benefit ongoing mental health treatment with high personalization and adaptability (2). Examples include monitoring for depression (3) and Alzheimer’s disease (4). Technology has the potential to provide a feasible lower-cost alternative to routine follow-up visits, with fewer constraints on patient scheduling and increased access to on-demand care triggered by sensor devices and provided remotely through telehealth platforms (5). However, with a wide range of commercial design specifications and intended usage scenarios, the cost-effectiveness of this technology remains uncertain.

In this paper, we used chronic depression as a case study and explored under what conditions a hypothetical remote monitoring technology can be cost-effective for managing ongoing psychiatric treatment. Depression is a complex and dynamic mental disorder characterized by emotional and physical symptoms that may result in disability, reduced quality of life and productivity, and increased risk of death. In the year 2019, 7.8 percent of all American adults had at least one major depressive episode, and 4.7 percent had regular feelings of depression (6;7). Depression is often unrecognized and untreated, and even once treatment begins it is often difficult to monitor its effectiveness (8). Treatment guidelines from several medical institutions suggest a follow-up frequency of at least every 12 months for patients on maintenance therapy to prevent the recurrence of major depression, and modifying treatment after a minimum of 4–6 weeks for patients with insufficient response to treatment (9–12). How to optimally schedule follow-up care for patients with partial response or to prevent relapse remains a significant challenge.

Remote depression monitoring technology can enable personalized interventions by adaptively scheduling follow-up visits, leading to timely treatment modification. For example, a mobile app, text messaging, or web site can prompt patients to complete a periodic (often bi-weekly) depression assessment, and a remote licensed therapist can review new symptoms, give feedback, and schedule an in-person follow-up if necessary (13). The patient’s health data are continuously collected to develop a personalized depression trajectory, and deviations can automatically generate an alert (14). Moreover, digital healthcare platforms can empower patients to monitor their health conditions and enable clinicians to address treatment failures much sooner than fixed-frequency medical follow-up (13). Research has also suggested that implementing measurement-based care (15), however, data are collected, can improve treatment effectiveness for major depression (16).

Cost-effectiveness analysis (CEA) is an economic evaluation tool to systematically investigate the costs and outcomes of comparable healthcare interventions (17;18). It provides a way for decision makers to use empirical data to best allocate scarce resources by estimating an incremental cost-effectiveness ratio (ICER) and comparing this ratio to a willingness-to-pay (WTP) threshold (18;19). Combined with decision-analytic models and simulation methods, CEA has been used to evaluate screening and treatment routines for depression (20–22). It also has been used to assess monitoring strategies for depression (23), diabetes (24), HIV (25), asthma (26), and hypertension (27).

We designed a novel decision-analytic model to evaluate the cost-effectiveness of remote monitoring technology for optimal depression treatment follow-up. Our method contributes by defining a multi-period Markov state to outline health levels and the short disease trajectory, guiding treatment modifications based on the state interpretation, using one-step health boosts to simulate treatment effects, and conducting sensitivity analyses for technology development. We hypothesized that the technology could schedule a patient’s next outpatient visit adaptively by detecting changes in the patient’s depression severity. We compared the remote monitoring strategy to four traditional nonremote follow-up strategies. We hope our proposed technology assessment method can be extended to evaluate other remote monitoring technologies in advancing cost-effective psychiatric services (28). The CHEERS 2022 statement was used to guide reporting for this study.

## Methods

### Overview

We established the baseline depression progression of a patient cohort using a data-informed simulation and then simulated chronic depression patients’ disease progression for 2 years with treatment assignment under five monitoring strategies. We developed a decision-analytic Markov-cohort model and calculated the costs and QALYs accordingly. We used R and Python for the analyses.

### Patient cohort simulation

The data-informed simulation of depression progression was based on an Electronic Health Record (EHR) data set (29). The data set is drawn from the EHR of four U.S. health systems participating in the Mental Health Research Network (MHRN) (HealthPartners, and the Colorado, Washington, and Southern California regions of Kaiser Permanente). Cohorts are defined by age (base case, 45 years) and sex (base case, 69 percent female). One of the most common depression severity measurements is the Patient Health Questionnaire-9 (PHQ-9) (30), a self-administered questionnaire to diagnose depression. PHQ-9 scores range from zero to 27 with a higher score denoting higher severity. Research has demonstrated that, in addition to making criteria-based diagnoses of depressive disorders, the PHQ-9 is a reliable and valid indicator of depression severity. Its conciseness, combined with these qualities, makes the PHQ-9 a valuable tool in both clinical and research environments (31). Although it is not perfect, the PHQ-9 is frequently used as the measurement of depression severity in clinical practice guidelines for the management of major depressive disorder (32). The EHR data set includes longitudinal PHQ-9 scores between the years 2007 and 2012; it also includes age range, sex, observation time interval, and treatment status.

**Figure 1.**
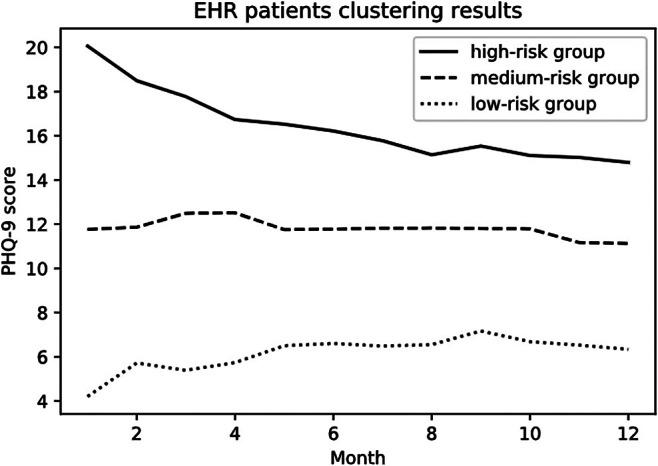
The average PHQ-9 score trajectories for each group.

We selected patients receiving ongoing treatment, which was defined as having had psychotherapy visits in the previous 90 days or filled prescriptions for antidepressants in the previous 180 days. We filtered patients having no fewer than six recorded PHQ-9 scores in an approximately one-year time window. This decision omitted 1069 (70 percent) of participants from the data set. We assigned twelve monthly periods for each patient by dividing their total days into 12 segments and calculated the mean PHQ-9 score for each month. The final data set contained 444 patients (307 female and 137 male). About 38 percent of the PHQ-9 scores were missing. We imputed missing data using the exponential weighted moving average (EWMA) (33) method to obtain all twelve monthly PHQ-9 scores for each patient. These scores depicted the baseline trajectories of depression progression. We clustered the 444 patients based on their PHQ-9 scores using the k-means clustering method (34). We converted each patient’s 12 PHQ-9 scores into a 12-dimensional vector and clustered them based on their Euclidean distances (35). Clustering results showed three groups determined by the severity of depression in the baseline trajectories: a high-risk group with 128 patients, a medium-risk group with 192 patients, and a low-risk group with 124 patients. We used the Silhouette method to identify the optimal number of clusters, evaluating each object’s similarity to its own versus other clusters; although the highest silhouette scores were obtained with two clusters, we chose three clusters to provide deeper insights, as the silhouette scores between two and three were close, and grouping into three risk categories – high risk, medium risk, and low risk – is both functional and intuitive. We used the trajectory in the clusters to simulate the baseline depression progressions for the patients with different severity of depression. We further classified depression severity into three levels based on the PHQ-9 score: healthy (H) with scores from zero to four; mild depression (M) with scores from five to nine; and moderate and severe depression (S) with scores from 10 to 27 (see Appendix Table 1). Note that severity is a grouping reflection of PHQ-9 scores; risk is a measure of how likely a patient was to have severe depression, and trajectory is a history of PHQ-9 scores. The mean trajectory of each risk group is shown in Figure 1. Although the trajectory of the high-risk group shows a decreasing trend in PHQ-9 scores, the scores still fall into the S level; thus the patients in this remain at high risk of experiencing severe depression.

### Disease progression simulation

We designed a decision-analytic Markov-cohort model with a monthly cycle to simulate chronic depression patients’ disease progression for 2 years. We defined a two-period combined Markov state with the patients’ depression level in the last and current month that captures a short-term trajectory that can be used to determine treatment response under an established treatment-switching strategy as shown in Appendix Figure 1. The states include HH, HM, HS, MH, MM, MS, SH, SM, and SS.

PHQ-9 scores greater than or equal to 10 have been found to be 88 percent sensitive and 88 percent specific for detecting major depression (30). Response to treatment is defined as a PHQ-9 score improvement of greater than 50 percent from baseline, and remission is defined as a PHQ-9 score of less than five maintained for at least 1 month (9). Based on these definitions, HM, MM, HS, MS, and SS are interpreted as nonresponse or relapse because these states represent staying in groups with scores of five or above (M or S) or moving to a worse level from *t* − 1 period to t period. HH, MH, SH, and SM show treatment responses because they involve remaining at or moving to a healthier level. Response states are further classified in two ways: (i) HH, MH, and SH stand for remission in which a patient has a PHQ-9 score less than five for at least 1 month; SM stands for a response without remission with some improvement in PHQ-9 score; (ii) SH and SM stand for unstable improvement because the patient was in S at the previous month; HH and MH stand for stable remission, in which a patient maintains a PHQ-9 score less than 10 for at least 2 months. We also assume that patients can die in any period regardless of their depression level.

Feasible state transitions are shown in Appendix Figure 1. The Markov state-transition diagram is shown in Appendix Figure 2. We estimated the baseline transition matrix based on numerical frequencies, counting all the transitions in the imputed EHR data set and calculating the transition probability from state a to state b (36). We estimated three separate transition matrices for the three risk groups (see Appendix Tables 2–4).

### Treatment assignment simulation

A traditional follow-up involves an outpatient clinical visit with the chance to change treatment. Remote monitoring indicates assessing depression severity remotely, triggering a visit for assessment and possible treatment change only when needed. We simulated nine treatment lines in total (37). A treatment line can consist of antidepressants alone or in combination with psychotherapy. Patients who failed to respond to the current treatment or relapsed can change to the next treatment line at each scheduled follow-up; these include patients who are in the HM, MM, HS, MS, or SS state. We modeled the treatment effect to be a one-period boost in health (38), represented by an increased probability of transitioning to a healthier state. Specifically, at the time of a treatment change, patients receive an additional probability (37) of transitioning to an H state in the current period (remission) compared to the baseline transition and an additional probability (37) of transitioning to an improved state in the current period. For example, at a follow-up point, a patient in the SS state may move to SH or move to SM. If the remission rate of that treatment line is pr_rm_, the response (excluding remission rate) is pr_rsp_, and the original proportion of state SS is p_ss_; then after the treatment boost, a proportion of pr_rm_ 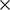 p_ss_ of the cohort will transition to state SH, and a proportion of pr_rsp_ 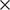 p_ss_ will transition to state SM. Afterward, the patient reverts to the baseline transition matrix until the next treatment change. To leave sufficient time for treatment response, per consensus guidelines for treatment of depression, there are no consecutive treatment changes in 2 months in our simulation (37;39). We assumed if a patient fails all nine treatment lines, then he/she receives no more health boost from treatment and returns to their baseline progression.

### Decision-analytic model

We compared five strategies: (i) adaptive remote monitoring technology with a false-negative rate of missing the next needed follow-up and a false-positive rate of an unnecessary follow-up. A perfect adaptive monitoring technology with 100 percent sensitivity and 100 percent specificity can immediately follow up patients in the nonresponse or relapse states who need a treatment change. (ii) rule-based follow-up strategy, which assigns a follow-up in 2 months for patients in states HM, MM, HS, MS, or SS; in 4 months for patients in state SH or SM; in 6 months for patients in state HH or MH. (iii–v) Fixed-frequency follow-up strategy regardless of patients’ health states. We evaluated the fixed two-month, four-month, and six-month follow-ups. After exhausting all nine treatment lines, patients in the rule-based and fixed-frequency strategies are assigned a six-month follow-up frequency (9–12). Remote monitoring patients who exhaust all nine treatment lines are assumed to continue monthly monitoring with no scheduled follow-up. We focused the investigation on how cost-effective the remote monitoring technology is compared to the rule-based follow-up strategy, which is the closest to the current practice according to the depression guidelines (9–12).

**Figure 2.**
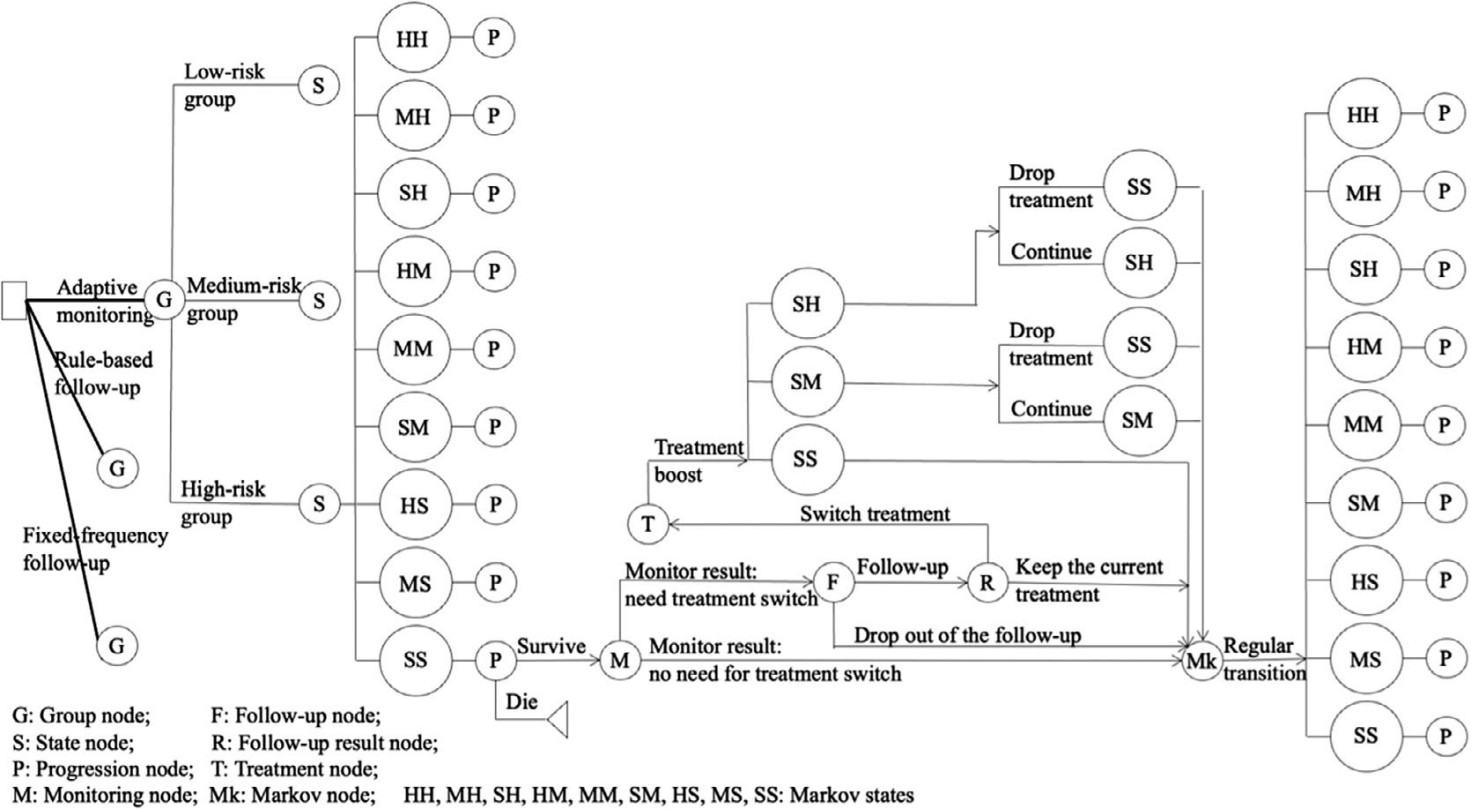
Decision-analytic model of depression monitoring and treatment simulation.

The initial states of the Markov model are matched with the group-specific initial health state distribution using the combination of severity levels in the first and second months in the clustered EHR data as shown in Appendix Tables 5–7. We simulated death at the beginning of each month. If it is not a follow-up month, patients progress according to their group-specific transition matrix. If it is a follow-up month, some patients may drop out of the follow-up. During a follow-up appointment, patients may change treatment. After changing to a new treatment, patients may discontinue the treatment due to adverse events. We assumed it takes some time for the adverse event to happen; thus patients can only discontinue treatment after 1 month of being on the treatment. Note that we incorporated the possibility of irregular use of remote monitoring technology resulting in any missing observation as a reduction in the technology’s sensitivity, and this impact is considered in our subsequent sensitivity analyses. The decision-analytic model is shown in Figure 2.

### Data and sources

Table 1 shows model input parameter values. We applied an annual discount rate of 0.03. See Appendix 1 for detailed explanations.

**Table 1.** Model input parameter values

Variable	Base case (range)	Reference
**General input**		
Annual discount rate	0.03	(50)
**Cohort characteristics**		
Age	45 (30–64)	EHR (29)
Sex		
Female	0.69	EHR (29)
Male	0.31	EHR (29)
Annual background death probability		
Age 45	0.00225	(51)^a^
The mortality hazard ratio		
Severe depression (S)	1.59 (1.113–2.067)	(52)
Moderate depression (M)	1.52 (1.064–1.976)	(52)
Minimal depression (H)	1.45 (1.015–1.885)	(52)
Annual probability of follow-up discontinuation^b^	0.211 (0.069–0.309)	(37;53)
**Remote monitoring technology**		
Sensitivity	0.76 (0.64–0.95)	(29)
Specificity	0.74 (0.43–0.82)	(29)
**Treatment effectiveness**		
Annual probability of treatment discontinuation due to adverse event	0.249 (0.151–0.391)	(37;53)
Remission probability, per month		
Treatment 1–3	0.397 (0.321–0.478)	(37;54)
Treatment 4–6 (relative risk vs. treatment 1)	0.93 (0.86–1.00)	(37;54)
Treatment 7–9 (relative risk vs. treatment 1)	0.77 (0.70–0.85)	(37;54)
After treatment 9	0	(37;54)
Response probability, per month		
Treatment 1–3	0.631 (0.553–0.703)	(37;54)
Treatment 4–6 (relative risk vs. treatment 1)	0.77 (0.73–0.81)	(37;54)
Treatment 7–9 (relative risk vs. treatment 1)	0.48 (0.44–0.53)	(37;54)
After treatment 9	0	(37;54)
**Costs, 2023$**		
Remote monitoring, per month^c^	12 (0–24)	(47;55)
Follow-up appointment, per time	131 (89–176)	(56)
Background treatment, per month^d^		
Treatment 1–3	1532 (1435–1628)	(57)
Treatment 4–6	1679 (1526–1831)	(57)
Treatment 7–9	1794 (1557–2030)	(57)
After treatment 9^e^	1669 (1505–1829)	(57)
Drug, per month	57 (18–147)	(58;59)
**Health utility**		
Level S^f^	0.49 (0.46 to 0.53)	(60)
Level M	0.62 (0.58 to 0.65)	(60)
Level H	0.7 (0.67 to 0.73)	(60)

a Sex difference is considered in the mortality rate, and the base case value is weighted by age proportion.

b We computed the annual follow-up discontinuation probability to be the total discontinuation probability subtracted by the drug adverse event discontinuation.

c The cost of remote monitoring is designed as a subscription or on-demand call fee, with the follow-up costs listed separately and not included in this remote monitoring cost.

d Background treatment cost includes the drug cost.

e The cost after treatment nine is computed as the average of treatment line 1–9.

f The utility of level S is the average utility based on the PHQ-9 score range (60).

### Analysis

Outcomes include total discounted costs, quality-adjusted life-years (QALYs) gained, and ICERs of the five strategies. We used the 2023 GDP per capita in the U.S., $81,630, as the WTP threshold (40). In the base case, we investigated which strategy is cost-effective and found the frontiers among all five strategies in each group. We further carried out sensitivity analysis on technology factors while keeping all other parameters at base case value to investigate under what ranges of sensitivity, specificity, and cost the remote monitoring technology is cost-effective compared to the rule-based strategy. We then kept the sensitivity, specificity, and cost of the remote monitoring technology at base case value and performed a deterministic sensitivity analysis for all the non-technology-related parameters in one-way, two-way, and scenario analyses.

Note that we did not include a probabilistic sensitivity analysis (PSA) in our study. PSA involves simultaneously varying multiple or all parameters using their respective uncertainty distributions to assess the robustness of model results. We did not include a PSA due to the hypothetical nature of the evaluated technology, which we felt putting detailed distributional assumptions on all model parameters would be somewhat arbitrary and give false confidence in the robustness. Moreover, because we already conducted extensive one-way, two-way, and multi-way sensitivity analyses, we do not think adding a PSA would provide significant additional insights.

## Results

### Base case

**Figure 3.**
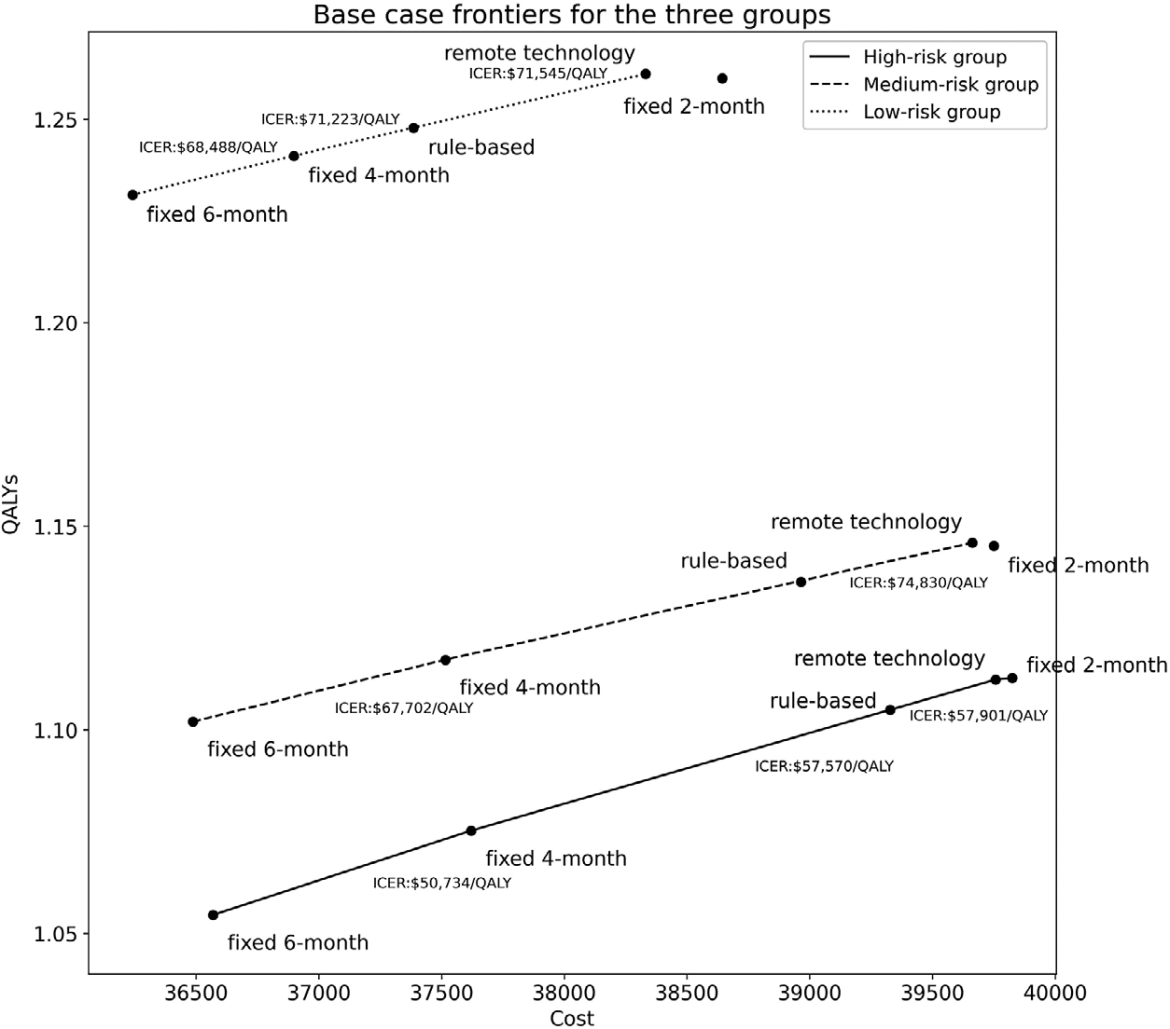
Base case cost-effectiveness frontiers for the three risk groups.

The costs, QALYs, and ICERs of the five strategies for each risk group are shown in Appendix Tables 9–11. The adaptive monitoring strategy has an ICER of $57,901/QALY, $74,830/QALY, and $71,545/QALY compared to the next best alternative for the high-risk, medium-risk, and low-risk groups, respectively. For the high-risk group, only the fixed frequency 2-month follow-up strategy has an ICER exceeding $81,630/QALY. For the medium-risk group, the fixed frequency 6-month, 4-month follow-up, and adaptive technology are not dominated by other strategies (i.e., a strategy with lower QALYs and higher cost compared to another strategy, or a linear combination of other strategies are dominated). For the low-risk group, fixed frequency 2-month is the only dominated strategy by remote monitoring technology. ICER frontiers are shown in Figure 3.

### Sensitivity analysis of technology factors

We quantified the impact of sensitivity, specificity, and monitoring cost on the cost-effectiveness of adaptive technology compared to the rule-based strategy, which resulted in 11*11*3*3 = 1089 scenarios as shown in Appendix Table 12. The technology could be (i) dominated by the rule-based follow-up strategy, which means the QALYs of the technology are less than the QALYs of the rule-based strategy; (ii) cost-saving, which means the technology has higher or equal QALYs and a lower cost compared to the rule-based strategy; (iii) with an ICER value. We are interested in the regions where the technology could be cost-saving or cost-effective with an ICER below $81,630/QALY.

**Figure 4.**
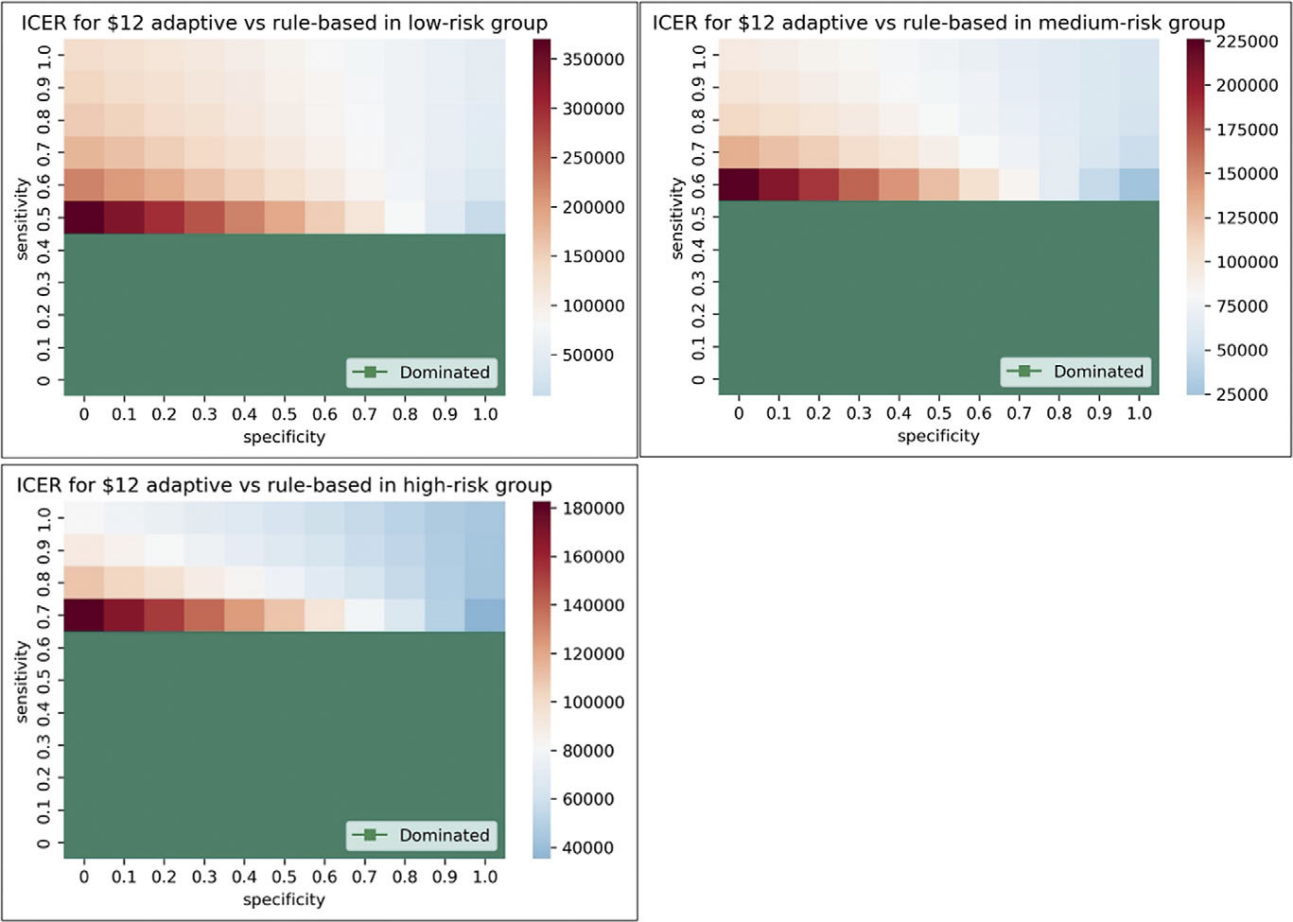
ICER for the technology versus rule-based strategy under $12 per month in three groups.

We used heat maps to show these results (Figure 4) for a fixed technology cost of $12 per month. Results for all settings are shown in Appendix Figures 3–11. The heat map is used to visualize data in two dimensions by color intensity (41). The *x*-axis shows specificity from zero to one, and the *y*-axis shows sensitivity from zero to one. We used the white color to represent the ICER values near the WTP threshold of $81,630/QALY. The red color means the ICER is above the threshold, which is not cost-effective; the blue color means the ICER is below the threshold, which stands for cost-effective. Results showed that within the same risk group, once the sensitivity reaches above a certain threshold, the adaptive technology is no longer dominated by the rule-based strategy. In the nondominated region, remote monitoring technology is more cost-effective at higher specificity and lower monitoring costs. Achieving high specificity is more important when the remote monitoring cost is high, and the technology could be cost-saving when the cost is free (or extremely low, see Appendix Figures 3, 6, and 9). The increase in cost sharply increases the requirement of sensitivity and specificity for the technology to be cost-effective. Comparing the results of the three groups, the technology required a higher sensitivity for sicker patients to outperform the rule-based strategy and is more cost-effective if it reaches the desired sensitivity in the higher-risk group.

### Sensitivity analysis on all parameters

We further performed a deterministic sensitivity analysis for all parameters not related to the technology itself in one-way, two-way, and multi-way analyses (detailed combinations provided in Appendix Table 13). We simulated 291 scenarios in total and 97 scenarios (including the base cases) for each group. For one-way sensitivity analysis (see details in Appendix 2), group difference appears in the follow-up cost: The technology is more cost-effective at a higher follow-up cost in the high-risk group, whereas it is more cost-effective at a lower follow-up cost in the low-risk group. The rule-based strategy assigns very frequent follow-ups for high-risk patients, but very few follow-ups for low-risk patients. This result implies that when patients are sicker and need more frequent follow-ups, the rule-based strategy is very aggressive in scheduling, resulting in some unneeded follow-up visits (false positives), and thus remote monitoring becomes more attractive when the cost of follow-up is high. However, for the healthier group of patients who do not need frequent follow-up, the remote technology may assign more unnecessary follow-ups while the rule-based strategy is already performing well. Therefore, remote monitoring becomes more attractive under lower follow-up costs compared to rule-based strategy.

The adaptive technology is cost-effective in 74 percent (72 out of 97) of the scenarios for the high-risk group, 67 percent (65 out of 97) of the scenarios for the medium-risk group, and 74 percent (72 out of 97) of the scenarios for the low-risk group. Under various parameter combinations, the significant parameters found in the one-way sensitivity analysis remain the key factors. Thus, we conclude that adaptive remote monitoring technology is generally cost-effective compared to the rule-based strategy and is more robust for high-risk and medium-risk groups. In addition, technology-related factors (cost, sensitivity, and specificity) are the main drivers of cost-effectiveness compared to other treatment and health-service-related parameters.

## Discussion

We assessed the cost-effectiveness of a hypothetical adaptive remote monitoring technology with variable accuracy and cost compared with a rule-based follow-up strategy that is similar to current practice as well as three fixed-frequency follow-up strategies. We simulated a cohort of chronic depression patients undergoing treatment for 2 years using a decision-analytic Markov-cohort model with nine available treatment lines.

We found remote monitoring technology is robustly cost-effective with appropriate technology factors: First, for the technology to be not dominated by another strategy, its sensitivity needs to reach a certain threshold, which increases with the patient’s baseline risk of severe depression. In addition to reaching a sensitivity threshold, the next priority is to improve specificity. Second, the most cost-effective technology does not align with perfect sensitivity, rather it is at a combination of high sensitivity and perfect specificity where the technology could be cost-saving or very cost-effective. This implies that false positives are very important factors to consider when designing a remote monitoring technology to avoid costly over-intervention. The cost of the technology can be higher only if both sensitivity and specificity are sufficiently high. Third, given high accuracy, the technology can be cost-effective under a variety of disease and treatment conditions. The technology is more cost-effective for sicker patients, lower cost for further treatment lines, higher treatment effectiveness, and poorer quality of life for severe depression. Fourth, patients may benefit more from the technology when the cost of follow-up is high. The technology could potentially help with the problems with the financing system that make outpatient follow-up visits too expensive. Please note that our conclusions regarding the cost-effectiveness of remote monitoring technology are based on our chosen WTP threshold of $81,630, in line with the GDP-based threshold. Different threshold choices may lead to varying conclusions.

Our findings on remote monitoring technologies being generally cost-effective align with previous research on the economic evaluations of remote monitoring strategies for chronic diseases. For example, one study highlighted the cost-effectiveness of telemonitoring for chronic obstructive pulmonary disease (COPD), showcasing its potential to reduce mortality and healthcare costs (42). Another study focused on Home Blood Pressure Telemonitoring and Case Management for hypertension care, demonstrating its effectiveness in improving care without increasing overall medical costs (43). Similarly, a study comparing the costs of home blood pressure telemonitoring with conventional office monitoring found telemonitoring to be more costly but still provided valuable insights into its cost-effectiveness (44). Additionally, a comparison of telemonitoring versus usual care for uncontrolled blood pressure management revealed that although telemonitoring was more effective, it also incurred higher costs (45). Furthermore, findings from another study emphasized the cost-effectiveness of remote monitoring for major adverse cardiovascular events in high-risk postmyocardial infarction patients (46). Overall, these studies collectively support the idea that remote monitoring holds promise as a cost-effective strategy for managing chronic diseases, despite some uncertainties, which aligns with our findings.

Note that patients with more severe depression are likely to have more frequent visits. Therefore, our filtering method that includes only patients with at least six PHQ-9 scores in 12 months may introduce a selection bias in the simulated cohort. Based on our results for the three risk groups, we observed that remote monitoring technology is more cost-effective in the high-risk group. Thus, we infer that the bias from our filtering method is likely to overestimate the cost-effectiveness of the monitoring technology if applied uniformly to all groups. Nonetheless, we believe it is reasonable to focus on patients with more severe depression when discussing the development of technology, as these patients are more critical in chronic disease management.

We considered sensitivity, specificity, and cost as important technology factors in evaluating remote monitoring technologies. However, variations in other technical aspects, such as user interface design, convenience, and patient preference, can lead to differences in patient adoption rate even with the same sensitivity and specificity. For example, quality of the user experience can influence the probability of patients discontinuing remote monitoring. Although this may not be a direct technological factor, it is a vital design consideration during technology development. We conducted a sensitivity analysis on factors related to discontinuation because an increase in discontinuation rate can be a proxy for poor user experience. Additionally, a more complex mode of administration may result in higher technology costs. We also performed a sensitivity analysis on the cost of the technology that can account for this variability.

To establish the base case sensitivity and specificity of the technology, we derived estimates from a previous study (29) that compared several machine-learning-based chronic depression monitoring algorithms. We selected the sensitivity and specificity of the best-performed method (29) as our base case. In the sensitivity analysis for technological factors, we explored the full range of sensitivity and specificity from 0 to 1, making it applicable to a wide range of remote monitoring technologies. One example can be an automatic telephonic assessment (ATA) system (47), from which we estimated our baseline cost. This system integrates tasks and alerts to providers, potentially enhancing the quality of depression care and boosting provider productivity. It screens patients for early detection of depressive episodes and conducts monthly monitoring, including PHQ-9 assessments for symptom tracking. Monitoring calls are scheduled at varying frequencies based on the severity of PHQ-9 scores. Additionally, digital health platforms like Medixine and CareClix facilitate remote patient monitoring and virtual healthcare services. Patients input health data manually or through devices, communicate with healthcare providers, receive personalized care plans, and access educational resources. These platforms monitor data for anomalies, generate alerts, and streamline healthcare management for patients and providers.

The main limitation of this study is our reliance on simulated data. We made many assumptions in our simulated framework, such as how the disease would progress and how treatment would improve health (48). These assumptions need further validation from clinical trials and observational studies. However, these studies cannot always fully evaluate future possible scenarios and outcomes; they also take a long time and are expensive. Simulations serve to supplement trials and propose potential trial designs. We also assumed the PHQ-9 questionnaire results represent the true health state of the patients, although the sensitivity and specificity of the PHQ-9 questionnaire can be imperfect (49). We could have incorporated the sensitivity and specificity of the PHQ-9 instrument as two additional parameters in our model. However, because we already modeled the sensitivity and specificity of remote monitoring, adding those of the PHQ-9 would introduce one more layer that may be unnecessarily complex. Therefore, we consolidated them into a single layer of parameters for remote monitoring accuracy. The accuracy of the questionnaire should be taken into consideration in future studies or other gold-standard measurements should be used to represent the true health state. Also, note that because we did not perform a PSA, we cannot make statements about the probability of cost-effectiveness or the value of further research to reduce uncertainty based on this deterministic analysis.

Our proposed model may be adapted to evaluate the cost-effectiveness of various novel remote monitoring technologies for other psychiatric services. Contributions from our modeling method include defining a multi-period Markov state to describe health levels that contain enough information to establish a short disease trajectory, deciding on whether the patient needs treatment modification based on the interpretation of the Markov states and establishing a treatment assignment strategy accordingly, using a one-step boost in health levels to simulate treatment effect emphasizing remission and response rates, and conducting extensive sensitivity analyses on technology factors to guide technology development requirement. Also, our study used a Markov-cohort model that differentiated patients only into three risk groups. For future research, we could incorporate additional patient characteristics to represent a more diverse population, including various demographic factors. Furthermore, we can explore the integration of personalized prediction models for depression trajectory within the framework to enhance treatment change detection. Additionally, extending the simulation period could enable us to evaluate the long-term cost-effectiveness and sustainability of remote monitoring technologies beyond the two-year timeframe used in the current study.

## Conclusions

This study aims to propose a systematic technology assessment method to guide the development of emerging monitoring technologies used in chronic disease care management through integrated computational tools and decision-analytic modeling. We identified several requirements for remote monitoring technology to be a cost-effective way to deliver chronic depression care services.

## Supporting information

Sun et al. supplementary material Sun et al. supplementary material

## Supplementary material

The supplementary material for this article can be found at http://doi.org/10.1017/S0266462324004677.
