# Supplementary material for: A decision-analytic method to evaluate the cost-effectiveness of remote monitoring technology for chronic depression
Source: Int J Technol Assess Health Care. 2025 Jan 16;40(1):e79. doi: 10.1017/S0266462324004677 (PMC11811955; doi:10.1017/S0266462324004677)
Supplement: Sun et al. supplementary material [file S0266462324004677sup001.docx]

**Title:** A decision-analytic method to evaluate cost-effectiveness of remote monitoring technology for chronic depression

**Appendix:** A decision-analytic method to evaluate cost-effectiveness of remote monitoring technology for chronic depression

**Appendix 1: Details on Model Input Parameters**

***Background mortality and hazard ratio***

The annual background death probability is estimated from mortality rates by age and sex using the 2017 U.S. Centers for Disease Control (CDC) life tables (1). We computed a weighted mortality rate based on the sex distribution of our simulated population in the base case. Patients with depression have a higher risk of death, thus we multiplied the mortality rates by the hazard ratios of 1.59, 1.52, and 1.45 for depression levels S, M, and H, respectively (2). In the sensitivity analysis, we assumed a 70% lower bound and a 130% upper bound.

***Follow-up and treatment discontinuation***

We assumed there are two types of discontinuations: follow-up discontinuation and treatment discontinuation. They add up to be the total discontinuation (3, 4). Follow-up discontinuation stands for the probability that the patient has not gone to the follow-up appointment as scheduled. For the treatment discontinuation, we only considered drug discontinuation due to adverse events (3, 4).

***Remote monitoring technology***

Remote monitoring technology is not yet mature and there is no standardized performance evaluation. We used the sensitivity and specificity of the technology from Lin, et.al. (5) that compared several machine-learning-based chronic depression monitoring algorithms. We selected the sensitivity and specificity of the best-performed method (5) as our base case. In the sensitivity analysis, we first explored how the sensitivity and specificity of the technology would affect its cost-effectiveness under different monitoring costs for all three groups. We exhaustively tested all sensitivity and specificity ranging from 0 to 1 with a gap of 0.1.

***Treatment effectiveness***

While different treatments can have similar remission and response rates, existing studies have shown that treatment tends to be less effective as patients become sicker and undergo multiple treatment lines (3, 6). In this model, we used the same remission and response probability for every three treatment lines. We estimated the remission and response probability as 0.397 and 0.631 in the base case, respectively, for the 1^st^ treatment line and used the relative risk versus the first line to calculate the effectiveness for the remaining lines (3, 6). In the sensitivity analysis, we changed the treatment effectiveness in three ways: adjusting only the remission and response probability in the 1^st^ line; adjusting only the relative risk for the 2^nd^ -9^th^ lines; and adjusting both.

***Costs***

All costs were adjusted to 2023USD.

Since remote monitoring is a relatively new technology, we assumed it could be a smartphone app to survey PHQ-9 combined with a call for medical consultation or follow-up scheduling. Thus, we estimated the base case monitoring cost according to CPT codes 99441 and 98966 (7) to be $10 in the Year 2019. We estimated the upper bound to be $20 based on the remote physiologic monitoring CPT code 99453 (7). For the lower bound, we assumed free technology usage (8). We adjusted the cost to Year 2023.

We estimated the follow-up cost to be $110 from the 2019 CPT code 99214 and estimated the lower bound and upper bound from CPT code 99213 and 99215, respectively (9) in the Year 2019. We separated the follow-up appointment cost from the total health care cost which is used as the background treatment cost (10). We calculated the monthly drug cost based on commonly prescribed antidepressants such as fluoxetine and sertraline (11) and its current price (12) (see Appendix Table 8). When a patient drops out of treatment due to an adverse event, we subtracted only the drug cost from the background treatment cost. We used the same treatment cost for treatment lines with the same remission and response probability. The background cost increased with additional treatment lines, denoting the patients’ deteriorating general health conditions by failing multiple treatments (10). We adjusted the cost to Year 2023.

***Health utility***

We estimated the utility values based on the PHQ-9 score range assigned for each level. In the base case, the utility for level S, M, and H was 0.493, 0.62, and 0.7, respectively (13).

**Appendix 2: Detailed Results from One-way sensitivity analysis**

We varied the parameters to their lower and upper bound separately. For the treatment effects, since the remission rate and response rate for the 2^nd^-9^th^ treatment lines are measured as a relative risk to the first line, we varied them in two scenarios: changing only the first line remission and response rate or keeping the first line and changing the relative risks. The first way is interpreted as improving or weakening all treatment effects, while the second way examines the impact of increasing or decreasing the difference between treatment lines. We varied the remission rate, the response rate, and the background treatment cost for each line in the same direction.

Tornado plots of the ICERs for adaptive technology versus rule-based strategy in the three groups as shown in Appendix Figures 13-15. The majority of the ICERs are below $81,630/QALY, which means the technology is robustly cost-effective. In all groups, the two most significant parameters are background treatment cost increment through treatment lines and remission/response rate for all treatment lines, followed by the utility of severe depression. The remission/response reduction through treatment lines and drug cost are also important. Thus, remote monitoring technology is more cost-effective if patients spend less for further treatment after failures, if the treatment is more effective, and if patients suffer more from severe depression.


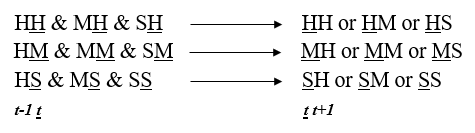


Appendix Figure 1: All feasible Markov state transitions


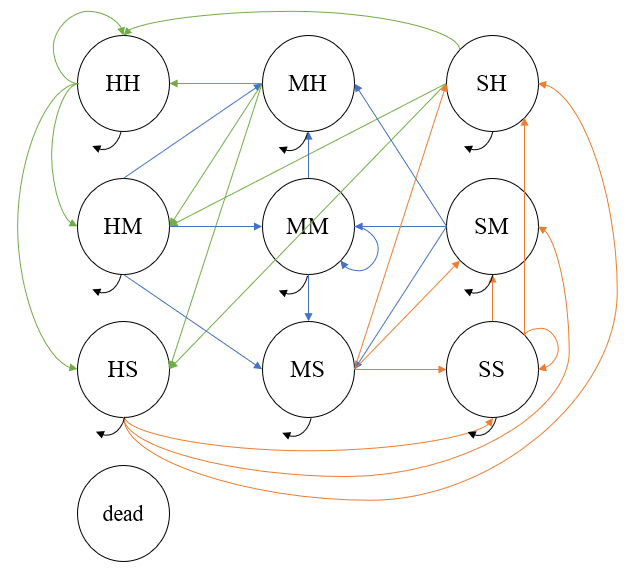


Appendix Figure 2: Overview of the Markov-cohort model to simulate depression progression

Appendix Table 1: Three depression level for the Markov model

| **Depression severity level** | **PHQ-9 score range** |
| --- | --- |
| H (healthy) | 0 - 4 |
| M (mild) | 5 - 9 |
| S (moderate & severe) | 10 - 27 |

Appendix Table 2: Transition matrix for the high-risk group

| To  From | HH | MH | SH | HM | MM | SM | HS | MS | SS |
| --- | --- | --- | --- | --- | --- | --- | --- | --- | --- |
| HH | 0.8333 |  |  | 0.1667 |  |  | 0.0000 |  |  |
| MH | 0.2941 |  |  | 0.6471 |  |  | 0.0588 |  |  |
| SH | 0.1333 |  |  | 0.4667 |  |  | 0.4000 |  |  |
| HM |  | 0.0588 |  |  | 0.6471 |  |  | 0.2941 |  |
| MM |  | 0.1343 |  |  | 0.7313 |  |  | 0.1343 |  |
| SM |  | 0.1250 |  |  | 0.2625 |  |  | 0.6125 |  |
| HS |  |  | 0.1111 |  |  | 0.0000 |  |  | 0.8889 |
| MS |  |  | 0.0625 |  |  | 0.1406 |  |  | 0.7969 |
| SS |  |  | 0.0098 |  |  | 0.0669 |  |  | 0.9233 |

Appendix Table 3: Transition matrix for the medium-risk group

| To  From | HH | MH | SH | HM | MM | SM | HS | MS | SS |
| --- | --- | --- | --- | --- | --- | --- | --- | --- | --- |
| HH | 0.7869 |  |  | 0.2131 |  |  | 0.0000 |  |  |
| MH | 0.3582 |  |  | 0.4478 |  |  | 0.1940 |  |  |
| SH | 0.1795 |  |  | 0.5641 |  |  | 0.2564 |  |  |
| HM |  | 0.2188 |  |  | 0.5313 |  |  | 0.2500 |  |
| MM |  | 0.0838 |  |  | 0.7614 |  |  | 0.1548 |  |
| SM |  | 0.1394 |  |  | 0.3990 |  |  | 0.4615 |  |
| HS |  |  | 0.1111 |  |  | 0.2963 |  |  | 0.5926 |
| MS |  |  | 0.0538 |  |  | 0.2097 |  |  | 0.7366 |
| SS |  |  | 0.0244 |  |  | 0.1388 |  |  | 0.8368 |

Appendix Table 4: Transition matrix for the low-risk group

| To  From | HH | MH | SH | HM | MM | SM | HS | MS | SS |
| --- | --- | --- | --- | --- | --- | --- | --- | --- | --- |
| HH | 0.8762 |  |  | 0.1158 |  |  | 0.0080 |  |  |
| MH | 0.5321 |  |  | 0.4312 |  |  | 0.0367 |  |  |
| SH | 0.3226 |  |  | 0.4194 |  |  | 0.2581 |  |  |
| HM |  | 0.2602 |  |  | 0.6098 |  |  | 0.1301 |  |
| MM |  | 0.1730 |  |  | 0.7075 |  |  | 0.1195 |  |
| SM |  | 0.2881 |  |  | 0.5254 |  |  | 0.1864 |  |
| HS |  |  | 0.2500 |  |  | 0.1875 |  |  | 0.5625 |
| MS |  |  | 0.0968 |  |  | 0.2258 |  |  | 0.6774 |
| SS |  |  | 0.0414 |  |  | 0.1862 |  |  | 0.7724 |

Appendix Table 5: Initial distribution for the high-risk group

| HH | MH | SH | HM | MM | SM | HS | MS | SS |
| --- | --- | --- | --- | --- | --- | --- | --- | --- |
| 0 | 0 | 0 | 0 | 0 | 0 | 0.0234 | 0.0469 | 0.9297 |

Appendix Table 6: Initial distribution for the medium-risk group

| HH | MH | SH | HM | MM | SM | HS | MS | SS |
| --- | --- | --- | --- | --- | --- | --- | --- | --- |
| 0 | 0 | 0 | 0.0260 | 0.1250 | 0.1302 | 0.0313 | 0.1198 | 0.5677 |

Appendix Table 7: Initial distribution for the low-risk group

| HH | MH | SH | HM | MM | SM | HS | MS | SS |
| --- | --- | --- | --- | --- | --- | --- | --- | --- |
| 0.3306 | 0.0968 | 0.1452 | 0.0645 | 0.2177 | 0.1452 | 0 | 0 | 0 |

Appendix Table 8: Monthly drug cost estimation in the Year 2019

| **Drug** | **Dosage (mg/day)** | **# Patients** | **% Patients** | **Average monthly cost** | **Weighted average cost** |
| --- | --- | --- | --- | --- | --- |
| Citalopram | 20 | 6,304 | 17% | 16.00 | 2.72 |
| Duloxetine | 60 | 4,460 | 12% | 123.47 | 14.86 |
| Bupropion | 300 | 4,364 | 12% | 64.32 | 7.57 |
| Sertraline | 50 | 4,173 | 11% | 27.59 | 3.11 |
| Fluoxetine | 20 | 3,631 | 10% | 21.23 | 2.08 |
| Escitalopram | 20 | 3,475 | 9% | 71.41 | 6.69 |
| Trazodone | 100 | 3,220 | 9% | 15.40 | 1.34 |
| Venlafaxine | 150 | 2,989 | 8% | 71.54 | 5.77 |
| Mirtazapine | 15 | 2,248 | 6% | 38.29 | 2.32 |
| Paroxetine | 20 | 2,201 | 6% | 22.24 | 1.32 |
| **Total** |  | 37,065 | 100% |  | 47.78 |

Appendix Table 9: Base case results for the high-risk group

| **Strategy** | **Cost, $** | **QALYs** | **ICER, $/QALY** |
| --- | --- | --- | --- |
| Fixed frequency 6-month | 36569 | 1.0545 |  |
| Fixed frequency 4-month | 37620 | 1.0752 | 50734 |
| Rule-based | 39328 | 1.1049 | 57570 |
| Remote monitoring technology | 39757 | 1.1123 | 57901 |
| Fixed frequency 2-month | 39825 | 1.1127 | 173366 |

Appendix Table 10: Base case results for the medium-risk group

| **Strategy** | **Cost, $** | **QALYs** | **ICER, $/QALY** |  |
| --- | --- | --- | --- | --- |
| Fixed frequency 6-month | 36487 | 1.1020 |  |  |
| Fixed frequency 4-month | 37515 | 1.1172 | 67702 |  |
| Rule-based | 38964 | 1.1363 | 75707 | extended dominated |
| Remote monitoring technology | 39662 | 1.1459 | 74830 |  |
| Fixed frequency 2-month | 39750 | 1.1452 | -127741 | dominated |

Appendix Table 11: Base case results for the low-risk group

| **Strategy** | **Cost, $** | **QALYs** | **ICER, $/QALY** |  |
| --- | --- | --- | --- | --- |
| Fixed frequency 6-month | 36240 | 1.2314 |  |  |
| Fixed frequency 4-month | 36898 | 1.2410 | 68488 |  |
| Rule-based | 37385 | 1.2479 | 71223 |  |
| Remote monitoring technology | 38331 | 1.2611 | 71545 |  |
| Fixed frequency 2-month | 38642 | 1.2600 | -283740 | dominated |

Appendix Table 12: Parameter settings in sensitivity analysis of technology factors

| **Factor** | **Number of levels** | **Levels** |
| --- | --- | --- |
| Sensitivity of the technology | 11 | [0, 0.1, 0.2, 0.3, 0.4, 0.5, 0.6, 0.7, 0.8, 0.9, 1.0] |
| Specificity of the technology | 11 | [0, 0.1, 0.2, 0.3, 0.4, 0.5, 0.6, 0.7, 0.8, 0.9, 1.0] |
| Cost of the technology, per month | 3 | [$0, $10, $20] |
| Group type | 3 | [High-risk, Medium-risk, Low-risk] |


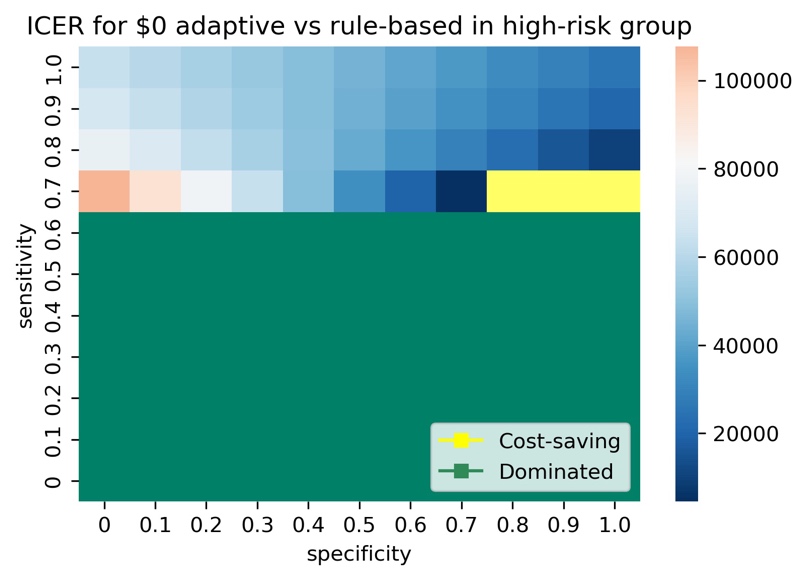


Appendix Figure 3: ICER for $0 adaptive remote monitoring technology versus rule-based follow-up strategy in the high-risk group


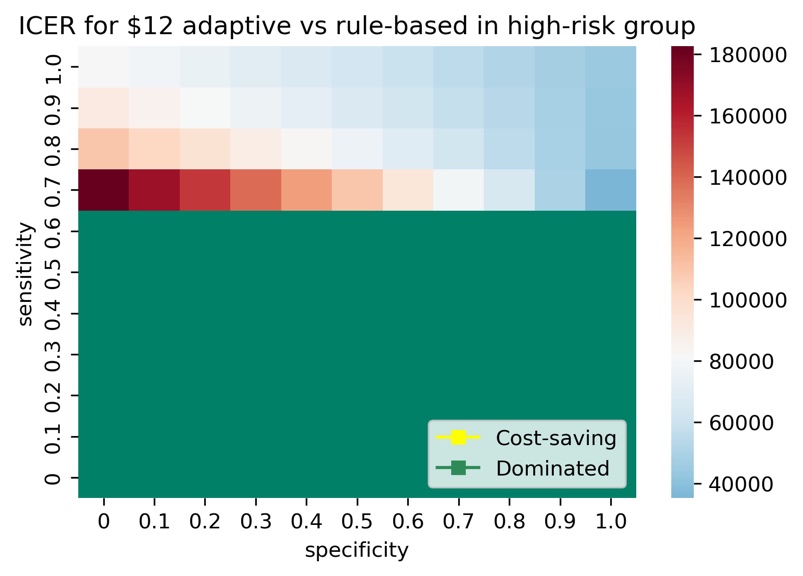


Appendix Figure 4: ICER for $12 adaptive remote monitoring technology versus rule-based follow-up strategy in the high-risk group


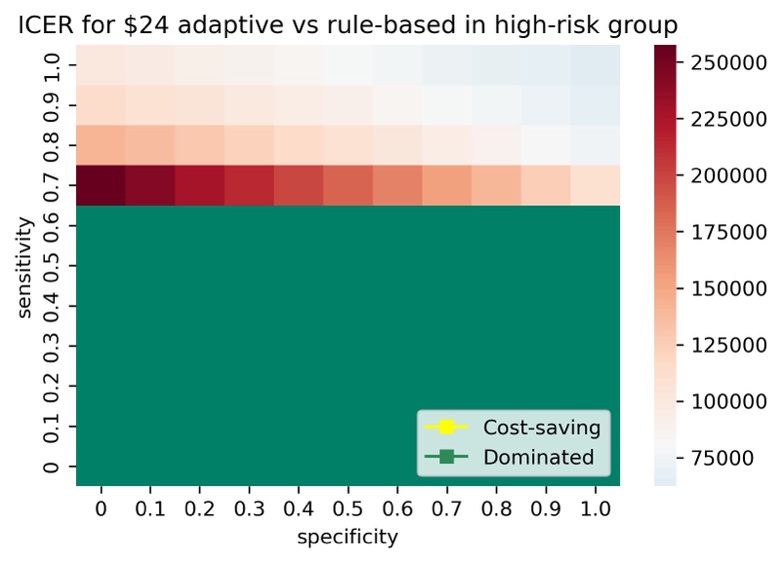


Appendix Figure 5: ICER for $24 adaptive remote monitoring technology versus rule-based follow-up strategy in the high-risk group


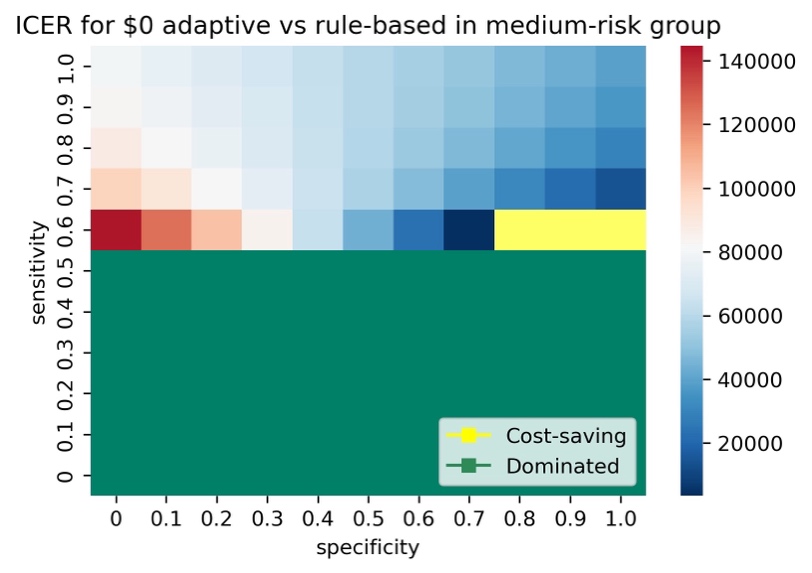


Appendix Figure 6: ICER for $0 adaptive remote monitoring technology versus rule-based follow-up strategy in the medium-risk group


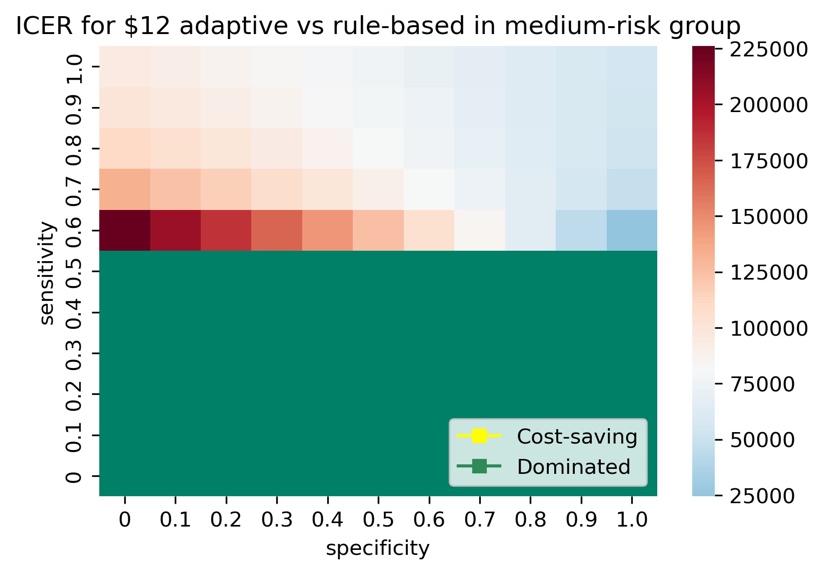


Appendix Figure 7: ICER for $12 adaptive remote monitoring technology versus rule-based follow-up strategy in the medium-risk group


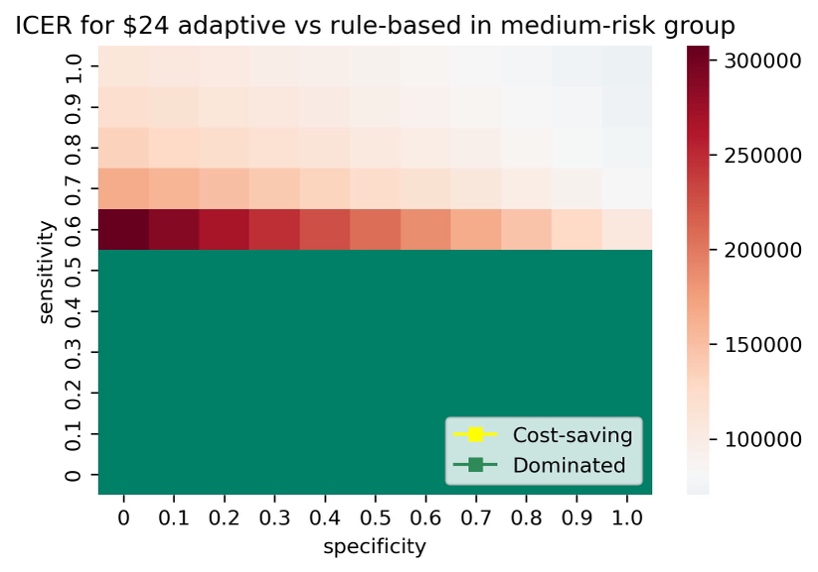


Appendix Figure 8: ICER for $24 adaptive remote monitoring technology versus rule-based follow-up strategy in the medium-risk group


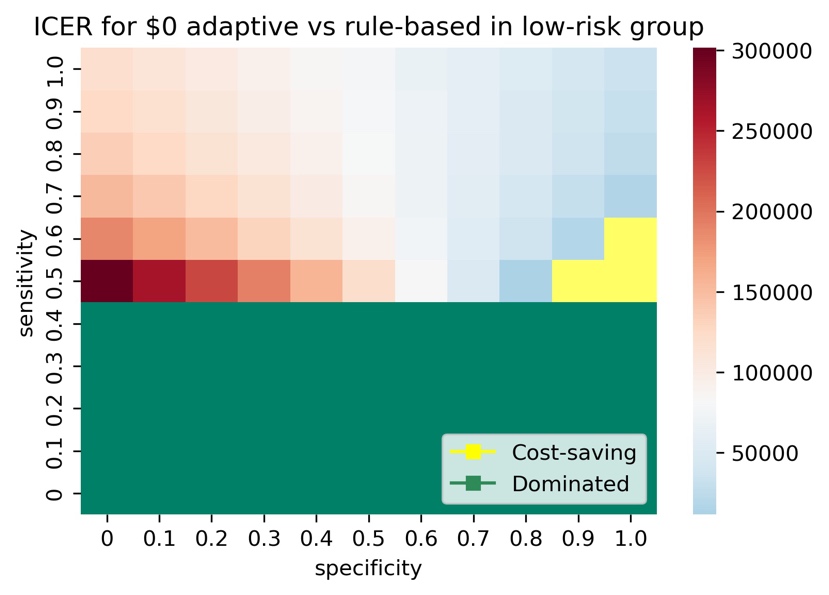


Appendix Figure 9: ICER for $0 adaptive remote monitoring technology versus rule-based follow-up strategy in the low-risk group


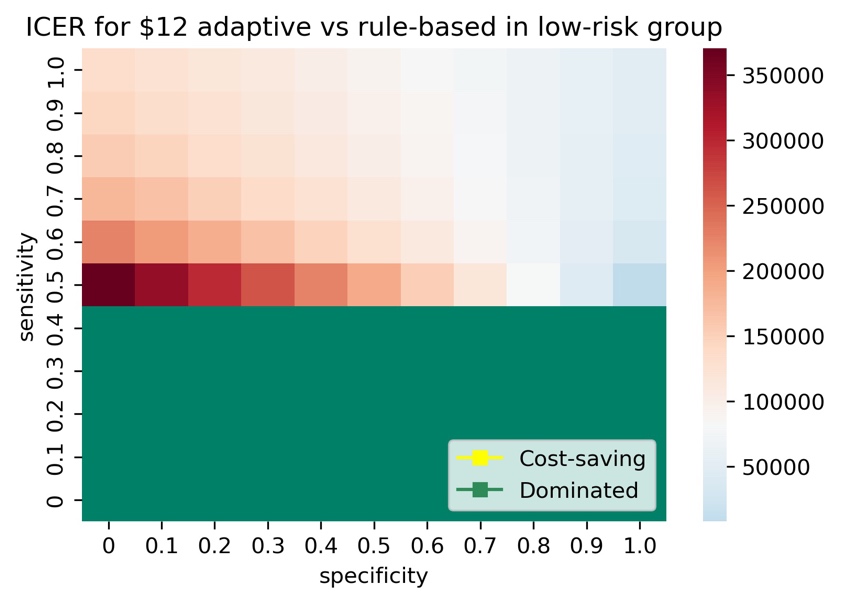


Appendix Figure 10: ICER for $12 adaptive remote monitoring technology versus rule-based follow-up strategy in the low-risk group


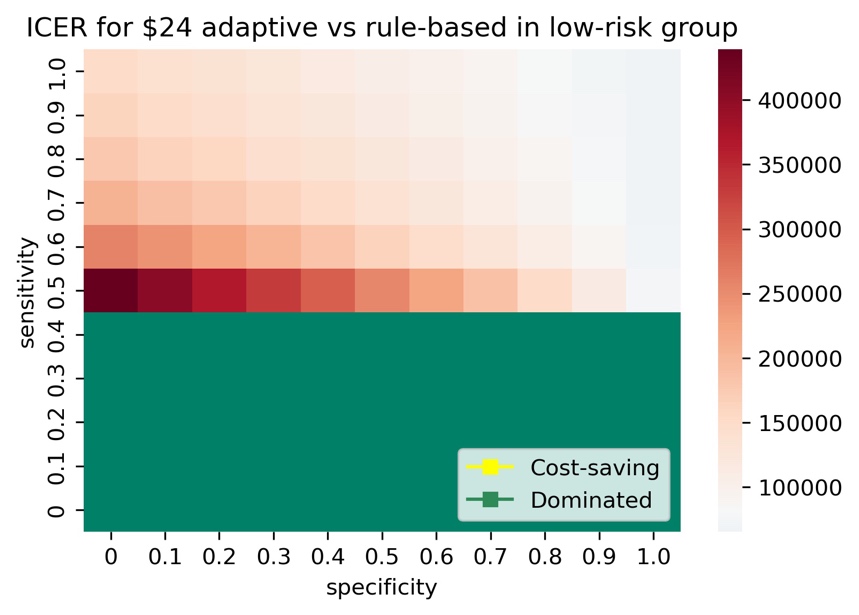


Appendix Figure 11: ICER for $24 adaptive remote monitoring technology versus rule-based follow-up strategy in the low-risk group


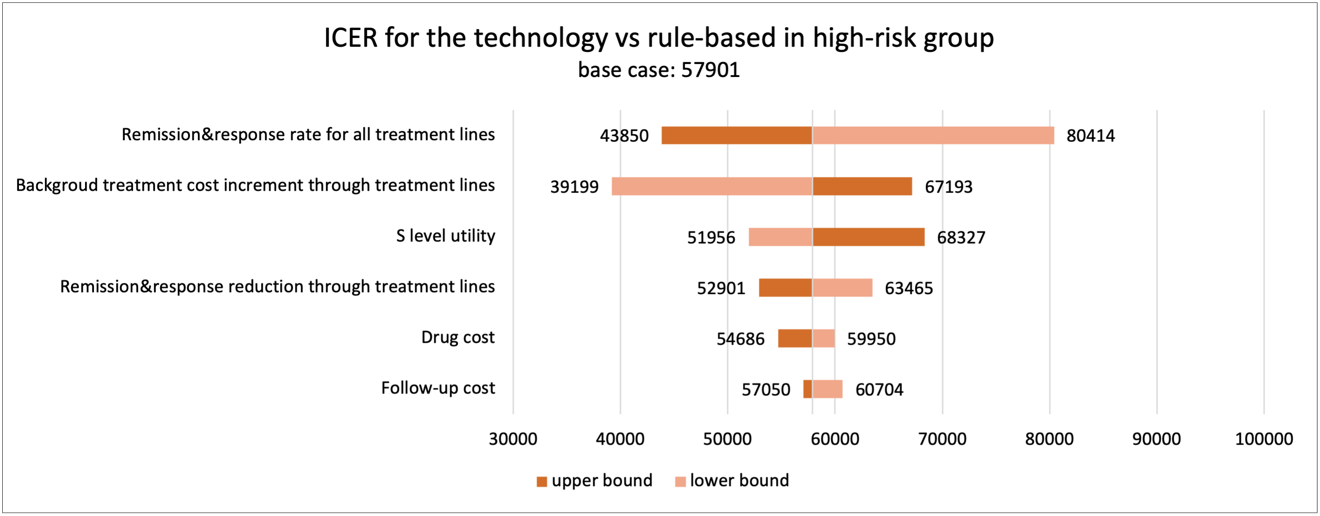


Appendix Figure 12: Tornado plot for one-way sensitivity analysis in the high-risk group


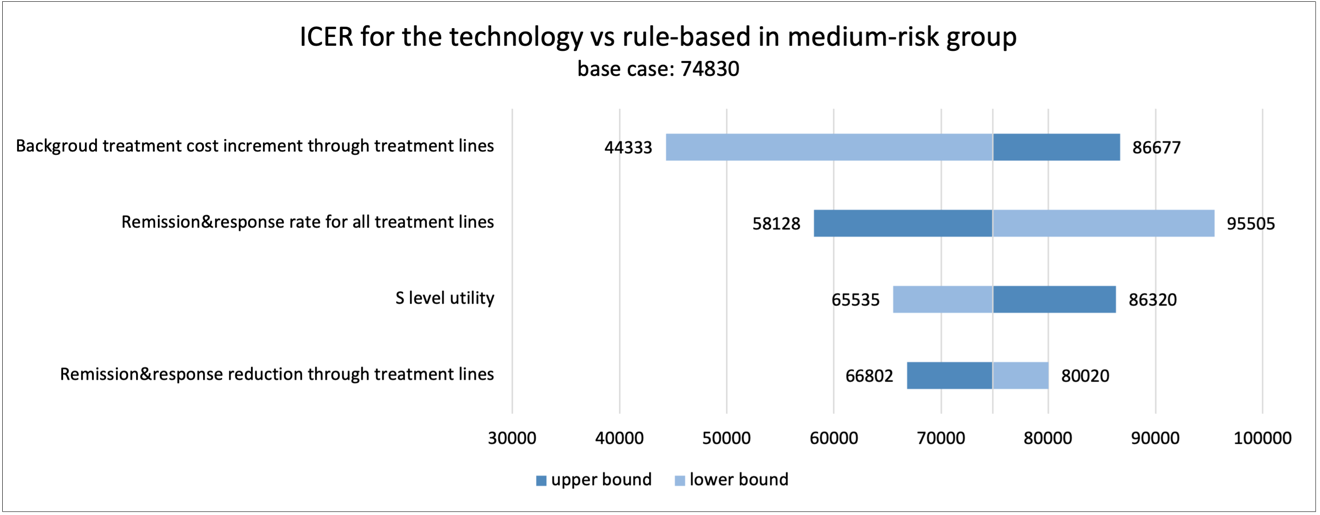


Appendix Figure 13: Tornado plot for one-way sensitivity analysis in the medium-risk group


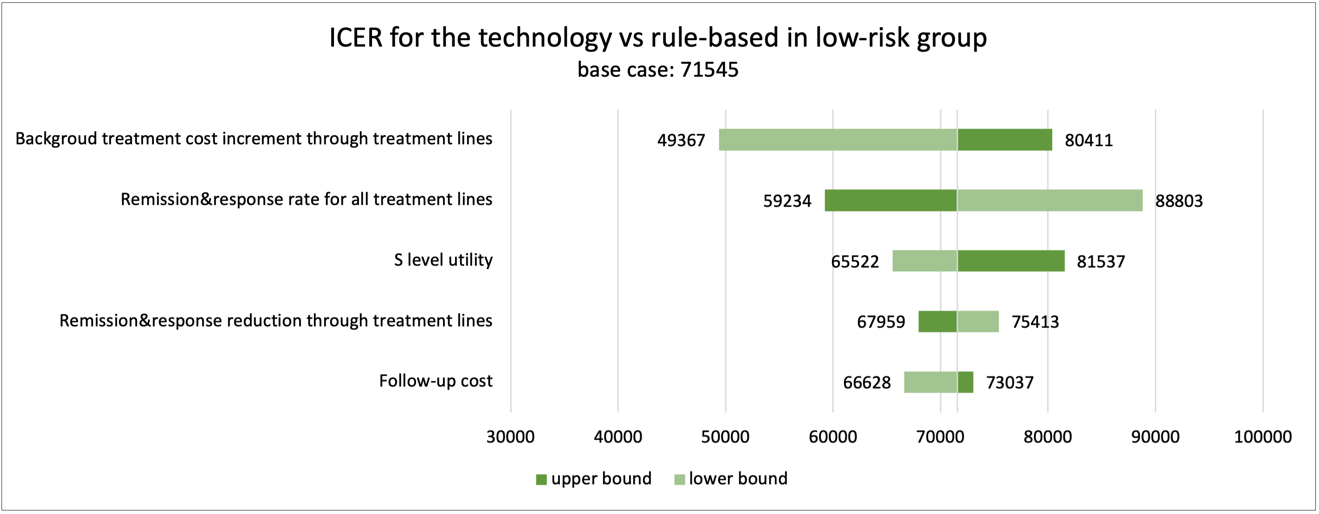


Appendix Figure 14: Tornado plot for one-way sensitivity analysis in the low-risk group

Appendix Table 13: Scenarios for two-way and multi-way sensitivity analysis

| **No.** | **Scenarios** |
| --- | --- |
| 1 | low follow-up discontinuation; low treatment discontinuation |
| 2 | high follow-up discontinuation; high treatment discontinuation |
| 3 | low all-line remission&response probability |
| 4 | high all-line remission&response probability |
| 5 | low all-line remission&response probability; low treatment discontinuation |
| 6 | high all-line remission&response probability; high treatment discontinuation |
| 7 | low all-line remission&response probability; high treatment discontinuation |
| 8 | high all-line remission&response probability; low treatment discontinuation |
| 9 | low all-line remission&response probability; low drug cost |
| 10 | high all-line remission&response probability; high drug cost |
| 11 | low all-line remission&response probability; high drug cost |
| 12 | high all-line remission&response probability; low drug cost |
| 13 | low 1st-3rd line remission&response probability; low treatment discontinuation |
| 14 | high 1st-3rd line remission&response probability; high treatment discontinuation |
| 15 | low 1st-3rd line remission&response probability; high treatment discontinuation |
| 16 | high 1st-3rd line remission&response probability; low treatment discontinuation |
| 17 | low 1st-3rd line remission&response probability; low drug cost |
| 18 | high 1st-3rd line remission&response probability; high drug cost |
| 19 | low 1st-3rd line remission&response probability; high drug cost |
| 20 | high 1st-3rd line remission&response probability; low drug cost |
| 21 | low 4th-9th line remission&response relative risk; low treatment discontinuation |
| 22 | high 4th-9th line remission&response relative risk; high treatment discontinuation |
| 23 | low 4th-9th line remission&response relative risk; high treatment discontinuation |
| 24 | high 4th-9th line remission&response relative risk; low treatment discontinuation |
| 25 | low background treatment cost; low follow-up cost |
| 26 | high background treatment cost; high follow-up cost |
| 27 | low background treatment cost; high follow-up cost |
| 28 | high background treatment cost; low follow-up cost |
| 29 | low background treatment cost; low drug cost |
| 30 | high background treatment cost; high drug cost |
| 31 | low background treatment cost; high drug cost |
| 32 | high background treatment cost; low drug cost |
| 33 | low follow-up cost; low drug cost |
| 34 | high follow-up cost; high drug cost |
| 35 | low follow-up cost; high drug cost |
| 36 | high follow-up cost; low drug cost |
| 37 | low 4th-9th line remission&response relative risk; low drug cost |
| 38 | high 4th-9th line remission&response relative risk; high drug cost |
| 39 | low 4th-9th line remission&response relative risk; high drug cost |
| 40 | high 4th-9th line remission&response relative risk; low drug cost |
| 41 | low background treatment cost; low follow-up cost; low drug cost |
| 42 | high background treatment cost; high follow-up cost; high drug cost |
| 43 | low background treatment cost; low follow-up cost; high drug cost |
| 44 | high background treatment cost; high follow-up cost; low drug cost |
| 45 | low background treatment cost; high follow-up cost; high drug cost |
| 46 | high background treatment cost; low follow-up cost; low drug cost |
| 47 | low utility; high follow-up discontinuation; high mortality hazard ratio |
| 48 | high utility; low follow-up discontinuation; low mortality hazard ratio |

**References:**

1. Life Table. Centers for Disease Control and Prevention, 2021

2. Cuijpers P, Vogelzangs N, Twisk J, et al.: Comprehensive meta-analysis of excess mortality in depression in the general community versus patients with specific illnesses. Am J Psychiatry 171:453-62, 2014

3. Ross EL, Vijan S, Miller EM, et al.: The Cost-Effectiveness of Cognitive Behavioral Therapy Versus Second-Generation Antidepressants for Initial Treatment of Major Depressive Disorder in the United States: A Decision Analytic Model. Ann Intern Med 171:785-95, 2019

4. Gartlehner G, Gaynes BN, Amick HR, et al.: Comparative Benefits and Harms of Antidepressant, Psychological, Complementary, and Exercise Treatments for Major Depression: An Evidence Report for a Clinical Practice Guideline From the American College of Physicians. Ann Intern Med 164:331-41, 2016

5. Lin Y, Huang S, Simon GE, et al.: Cost-effectiveness analysis of prognostic-based depression monitoring. IISE Transactions on Healthcare Systems Engineering 9:41-54, 2019

6. John Rush A, Trivedi M, Wisniewski S, et al.: STAR-D (2006; AjPsych) Tiered approach for depression. Am J Psychiatry 16311:1905-17, 2006

7. McManus M, White P, Schmidt A, et al.: 2020 coding and reimbursement tip sheet for transition from pediatric to adult health care. 2020

8. Wu S, Vidyanti I, Liu P, et al.: Patient-centered technological assessment and monitoring of depression for low-income patients. J Ambul Care Manage 37:138-47, 2014

9. Evaluation and Management Services. American Academy of Sleep Medicine

10. Russell JM, Hawkins K, Ozminkowski RJ, et al.: The cost consequences of treatment-resistant depression. J Clin Psychiatry 65:341-7, 2004

11. Trevino LA, Ruble MW, Trevino K, et al.: Antidepressant Medication Prescribing Practices for Treatment of Major Depressive Disorder. Psychiatr Serv 68:199-202, 2017

12. Drug Savings. GoodRx

13. Kolovos S, Bosmans JE, van Dongen JM, et al.: Utility scores for different health states related to depression: individual participant data analysis. Qual Life Res 26:1649-58, 2017
